# Icelandic herring-eating killer whales feed at night

**DOI:** 10.1007/s00227-016-3059-8

**Published:** 2017-01-30

**Authors:** Gaëtan Richard, Olga A. Filatova, Filipa I. P. Samarra, Ivan D. Fedutin, Marc Lammers, Patrick J. Miller

**Affiliations:** 1 Sea Mammal Research Unit, Scottish Oceans Institute, University of St Andrews, St Andrews, Fife, KY16 8LB UK; 2 Ecole Normale Supérieure de Lyon, 69007 Lyon, France; 3 Faculty of Biology, Moscow State University, Moscow, Russia 119234; 4 Marine and Freshwater Research Institute, Skulagata 4, 101 Reykjavík, Iceland; 5 Hawaii Institute of Marine Biology, Kaneohe, HI 96744 USA

## Abstract

Herring-eating killer whales debilitate herring with underwater tail slaps and likely herd herring into tighter schools using a feeding-specific low-frequency pulsed call (‘herding’ call). Feeding on herring may be dependent upon daylight, as the whales use their white underside to help herd herring; however, feeding at night has not been investigated. The production of feeding-specific sounds provides an opportunity to use passive acoustic monitoring to investigate feeding behaviour at different times of day. We compared the acoustic behaviour of killer whales between day and night, using an autonomous recorder deployed in Iceland during winter. Based upon acoustic detection of underwater tail slaps used to feed upon herring we found that killer whales fed both at night and day: they spent 50% of their time at night and 73% of daytime feeding. Interestingly, there was a significant diel variation in acoustic behaviour. Herding calls were significantly associated with underwater tail slap rate and were recorded significantly more often at night, suggesting that in low-light conditions killer whales rely more on acoustics to herd herring. Communicative sounds were also related to underwater tail slap rate and produced at different rates during day and night. The capability to adapt feeding behaviour to different light conditions may be particularly relevant for predator species occurring in high latitudes during winter, when light availability is limited.

**Electronic supplementary material:**

The online version of this article (doi:10.1007/s00227-016-3059-8) contains supplementary material, which is available to authorized users.

## Introduction

Investigating top predator behaviour is essential for a full understanding of the ecosystem they inhabit and the role that they play in it. Indeed, marine predator’s behaviours are influenced by diverse intrinsic and extrinsic factors. Prey abundance and distribution vary spatially within the water column, i.e. in depth, but also with time, either on short timescales, such as diel migration, or on longer scales, such as seasonal migration. Such diverse use of the water column by prey, both spatially and temporally, should influence the diving and foraging patterns and behaviour of their predators (e.g. Baird et al. 2005; Friedlaender et al. 2009; Arranz et al. 2011; Friedlaender et al. 2013; Samarra and Miller 2015). Day-night differences in light availability may also affect predator–prey interactions. For example, fish catchability may increase in the absence of light, either during night or at depth (Casey and Myers 1998). Thus, light availability could impact the foraging behaviour of marine predators. For example, Miller et al. (2010) revealed day-night differences in the diving behaviour of mammal-eating killer whales that were most likely explained by day–night ecological differences, such as differences in prey detectability due to ambient light or changes in prey behaviour.

Herring (*Clupea harengus*) is an important prey species for a number of marine predators, and it undertakes both diel and seasonal migrations. Throughout the year herring migrates between overwintering, spawning and feeding grounds (Holst et al. 2004) with concurrent changes in its behaviour, such as school size, preferred depth and density (Nøttestad et al. 2004). In addition, preferred depth also changes throughout the day, with a diel migration from deeper waters during the day to the surface layer during the night (Dommasnes et al. 1994; Huse and Ona 1996). In Iceland and Norway, killer whales (*Orcinus orca*) feed upon herring using a coordinated strategy to gather the herring and then slapping the prey ball with their tail to debilitate the fish (Similä and Ugarte 1993; Simon et al. 2005, 2007). These underwater tail slaps consist of multiple pulses over a short duration of ~300 ms with source levels of 186 ± 5.4 dB re. 1 μPa at 1 m across a broadband frequency range centred at 46.1 ± 22.3 kHz (Simon et al. 2005).

Killer whale groups produce unique and stable repertoires of stereotyped pulsed calls that differ between groups (Ford 1989, 1991) but are generally not specific to behavioural context (Ford 1989). From well-known populations, such as in the North Pacific, killer whale finer-scale groups have been described as matrilineal units, i.e. matrilines composed of an oldest-surviving female adult with several offspring generations (Bigg et al. 1990; Baird and Whitehead 2000; Ford et al. 2000). Matriline composition and interactions vary according to killer whale ecology. Indeed, optimal foraging group sizes depend on trade-offs between the ability to detect prey and the probability to be detected by potential prey (Baird and Dill 1996). Within a killer whale population, matrilines that associated at least 50% of the time were considered to form a ‘pod’ (Bigg et al. 1990). Matrilines in the same pod share a unique acoustic repertoire (Ford 1989, 1991) and are genetically more closely related than matrilines from different pods (Barrett-Lennard 2000). However, different pods can share a part of their repertoire, in which case they are considered part of the same acoustic ‘clan’ (Ford 1991). Yurk et al. (2002) revealed that two acoustic clans in Alaska are two maternal lineages, strengthening the idea of vertical maternal cultural transmission of vocal repertoires. Unique pulsed calls work as vocal signature, either matriline or pod or clan, and thus contain important information during social activity with other groups (Ford 1989, 1991; Deecke et al. 2000; Miller and Bain 2000), or to maintain cohesion while hunting (Miller 2002; Lammers and Au 2003).

During feeding, herring-eating killer whales increase the rate of production of communication sounds (Van Opzeeland et al. 2005; Samarra and Miller 2015), suggesting that acoustic communication may be used to coordinate whale movements and/or help herd the herring (Similä and Ugarte 1993; Simon et al. 2007; Shapiro 2008). Call production decreases when whales feed non-cooperatively upon herring discarded from fishing boats (Van Opzeeland et al. 2005), supporting the important role of acoustic communication during coordinated feeding. Thus, we might expect that variations in feeding behaviour in different ecological contexts will be reflected in differences in acoustic behaviour, but such variations are still poorly understood.

Herring-eating killer whales off Iceland produce a feeding-specific pulsed call thought to be aimed at prey and function as an acoustic means to herd the herring (‘herding’ call; Simon et al. 2006). Feeding-specific sounds thought to be directed at prey are also produced by bottlenose dolphins when feeding upon salmon (Janik 2000) and humpback whales when bubble-net feeding on herring (Cerchio and Dahlheim 2001). These calls are similar in structure to killer whale herding calls, suggesting convergence in acoustic behaviour that would facilitate the capture of herring (Simon et al. 2006). The production of feeding-specific sounds allows investigation of feeding occurrence, as well as variations with time of day or season, using passive acoustic monitoring (e.g. Schaffeld et al. 2016).

Herding calls of Icelandic killer whales have a high intensity (estimated source levels of 169–192 dB pp re 1 μPa @ 1 m; Simon et al. 2006), a low frequency (between 400 and 1400 Hz; Samarra 2015), a lack of frequency modulation and a long (~3 s) duration (Simon et al. 2006). Similar herding calls were also recorded from herring-eating killer whales in Shetland (Deecke et al. 2011). However, herding calls are not consistently produced in all feeding events (Simon et al. 2006; Samarra 2015), and it is not clear what factors drive its production. Variations in the production of the call and in the characteristics of calls produced may suggest that the herding call is group-specific (Simon et al. 2006; Samarra 2015); however, this has not been demonstrated to date.

In previous boat-based behavioural studies on herring-eating killer whales (e.g. Similä and Ugarte 1993; Simon et al. 2005, 2006, 2007), data collection was only possible during the daytime. When feeding during the day, the whales flash their white bellies to scare the fish, herding the herring school further, and therefore killer whales may depend on daylight to catch herring (Nøttestad et al. 2002). However, given the short length, i.e. between 4 and 6 h or less, of daylight during winter in high latitude areas, such as Iceland, it appears unlikely that feeding is limited to daylight time.

In this study we contrasted the acoustic behaviour of killer whales between day and night, using an autonomous acoustic recorder deployed in an Icelandic fjord during 1 month in winter 2014. Overwintering herring gather in large aggregations in fjords during the winter months (Óskarsson et al. 2009), and killer whales are known to feed on these herring. Using acoustically detectable underwater tail slaps as a proxy of feeding activity (Simon et al. 2005, 2007; Samarra and Miller 2015), we aimed to assess whether killer whales feed at night, and how acoustic behaviour related to feeding might differ between day and night.

## Materials and methods

### Data collection

**Fig. 1:**
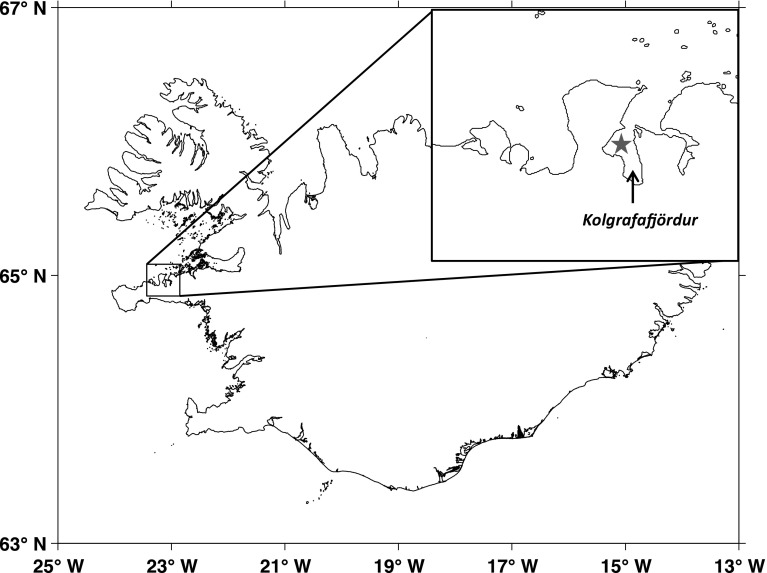
Field site (Kolgrafafjordur) with the location of the deployed hydrophone represented by a star

An Ecological Acoustic Recorder, EAR (Lammers et al. 2008), was deployed in Kolgrafafjörður, Iceland (64°57′N, 23°07′W, Fig. 1) for 37 days (from 22 February to 31 March) in winter 2014. This fjord was part of the overwintering grounds of the Icelandic summer-spawning herring stock in 2014 (ICES 2014). During the deployment, the EAR recorded 5 min of audio every 10 min at a sampling rate of 64 kHz, and used a Sensor Technology SQ26-01 hydrophone with a sensitivity of −193.5 dB (frequency response: ±1.5 dB from 1 Hz to 28 kHz). The small size of the fjord (approximate width of 2 km and length of 5 km) allowed us to consider that we would not miss high-intensity sounds (such as the herding call) produced by killer whales within this fjord.

### Acoustic processing

**Fig. 2:**
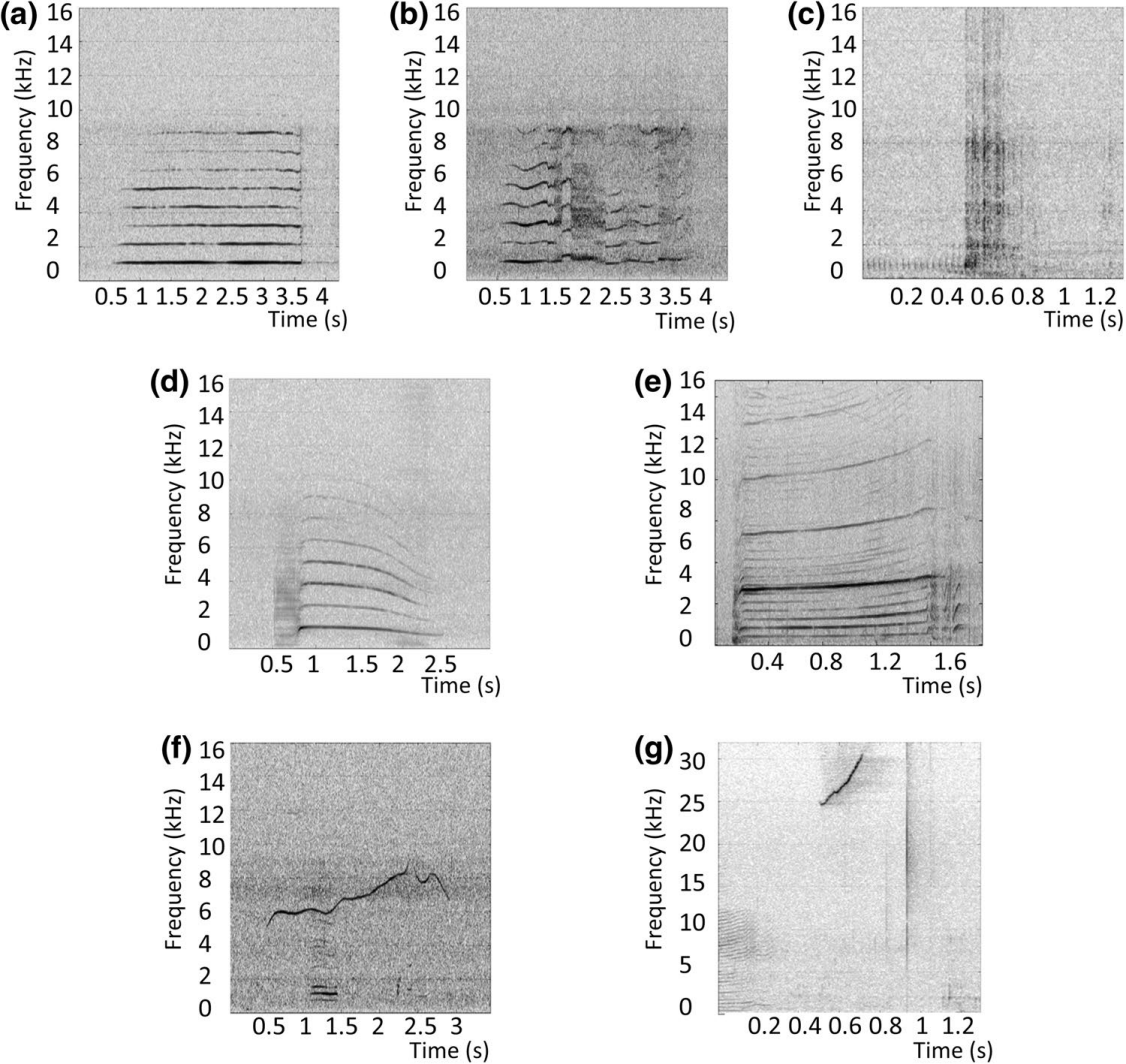
Examples spectrograms for each sound category, **a** linear herding call, **b** nonlinear herding call, **c** tail slap, **d** monophonic call, **e** two-voice call, **f** whistle and **g** high-frequency whistle. Spectrogram parameters: window = Hanning; FFT length = 2048; window length = 1024; overlap = 0.875

In order to reduce the dataset for analysis and to obtain representative samples across the entire recording period, we analysed the first minute of each 5-min file. The files were processed manually using Adobe Audition CS6 (version 5.0), by aural and visual inspection of spectrograms to detect killer whale sounds. Each sound was marked and then classified into one of seven categories (Fig. 2). Based upon consistent variations, we classified the ‘herding call’ into two categories: calls with high intensity (source level estimated at 169–192 dB pp re 1 μPa @ 1 m), low frequency (400–1400 Hz), lack of frequency modulation and a long duration (~3 s), similar to those described in earlier studies (Simon et al. 2006; Samarra 2015) were referred to as “linear herding calls” (Fig. 2a); herding calls that included nonlinear phenomena, such as frequency jumps, subharmonics or noise, as defined in Fitch et al. (2002) and Tyson et al. (2007), were referred to as “nonlinear herding calls” (Fig. 2b; Samarra 2015). Sounds with very short duration, i.e. around 300 ms, and with very large broadband frequency (up to the limit of the recording system) as described by Simon et al. (2005) were categorised as underwater tail slap (Fig. 2c). Pulsed calls consisting of a single frequency component, and which were not “herding” calls, were categorised as monophonic calls (Fig. 2d, Filatova et al. 2007) and those containing an overlapping of two independent frequency components were marked as biphonic calls (Fig. 2e, Miller 2002). Sounds based on a non-pulsed tonal format with a narrow-band tone above 4 kHz were categorised as whistles (Filatova et al. 2016), visually and aurally distinguishable from pulsed calls (Ford 1989; Riesch et al. 2006), with an approximately maximum frequency range 3–17 kHz (Fig. 2f, Thomsen et al. 2001), and those with fundamental frequency contours above 17 kHz, were classified as high-frequency whistles (Fig. 2g, Samarra et al. 2010). We chose these broad sound categories (i.e. monophonic calls, biphonic calls, whistles, high-frequency whistles and underwater tail slap) to avoid unnecessary variation caused by group-specific differences in repertoires within these broad sound categories. Day and night data were processed identically, and the observer classifying sounds was blind to the period of the day when sounds were recorded.

For each file, we summed the total number of sounds of each category. We then estimated the solar angle using time and spatial coordinates, using the function *solarpos* (package *maptools*) from the software R (R Development Core Team 2015) in order to define whether each file was recorded during the day, night or civil twilight. Solar angles are estimated from the horizon, so in theory they could vary between −90° and +90°. As we considered the civil twilight, we set this period between −6° and 0°. Thus, day is defined by positive solar angles, i.e. the sun is above the horizon, and night is defined as negative angles below −6°.

In order to ensure the detectability of sounds did not vary between day and night, we compared ambient noise levels on the recorder during daytime and night-time. Additionally, we used a proxy for recording quality comparable between daytime and night-time. Due to their high intensity (source level 169–192 dB pp re 1 μPa @ 1 m), long duration (~3 s) and low frequency (400–1400 Hz, Simon et al. 2006; Samarra 2015), we assumed that herding calls could be detected from a longer distance than sounds of other categories. Thus, we assessed the quality of all herding calls in the recording periods, as a proxy for overall recording quality, by establishing whether each herding call was masked by noise or could be clearly distinguished. For that purpose, we measured the root mean square (RMS) sound pressure level (SPL) values of the recorded waveform over one-third octave bands with a custom-written script in MATLAB (The MathWorks, Natick, MA, USA). An octave band filter has been applied to both the signal-plus-noise and the noise within the extracted marked sound. The process compared the RMS SPL (dB re 1 muPa^2^) of calls (with overlapping background noise) to the RMS SPL of the ambient noise (without any call) a few seconds before or after each call. Then we calculated the signal-to-noise ratio (SNR) as the difference of both RMS measures (call and noise). We considered calls to be of high quality if they had peak SNR > 10 dB in at least one of the third octave bands. Finally, we compared the proportion of high quality herding calls between day and night to assess whether there were differences in recording quality between day and night. Additionally, we compared the mean RMS SPL of the ambient noise between day and night within a 200 Hz–15 kHz band, by estimating the mean difference of random RMS noise levels between the two periods.

### Units of analysis

Killer whale presence in the fjord was assumed if any killer whale sound was marked within each 5-min acoustic file. Files with killer whale sounds appeared to occur in bouts. Absence of sounds could be due to true killer whale absence or because killer whales were present but not vocalising (e.g. travelling, Simon et al. 2007; Samarra and Miller 2015) or remained undetected by the recording equipment. Therefore, we conducted a bout analysis to determine the bout criterion interval (Slater and Lester 1982; Sibly et al. 1990), i.e. we aimed to objectively define a time interval threshold between files with sounds to establish a ‘presence event’. We plotted the log frequency of intervals between files with detected sounds using *cftool* in MATLAB and fitted the distribution with one- and two-process exponential models (Sibly et al. 1990). We observed that the best curve fit to the distribution of intervals was a two-process exponential model (*r* ^2^ = 0.95). We then minimised the total time misclassified to specify the threshold (Slater and Lester 1982; Miller et al. 2004) giving us a time interval threshold of 10.8 files, i.e. any two files with killer whale sounds separated by more than 10 files (approximately 110 min) without sounds were considered two different presence events.

Each presence event was then assigned to one of two periods: day or night. All cases where the presence event continued through day into night or vice versa (8 presence events), and included twilight were removed. By removing the twilight we removed the gradient of luminosity between day and night.

To provide an overview of variations in sound production with day/night we calculated the sound production rate (number of sounds produced per minute) for each presence event by dividing the total number of sounds from each category by the duration of the presence event. We also categorised each presence event as either a feeding event, if it contained at least one underwater tail slap, or a non-feeding event if it contained no underwater tail slaps. Thus, each presence event was either considered as a ‘feeding event’ or a ‘non-feeding’ event, allowing us to estimate the number of feeding events for day and night. Indeed, Simon et al. (2007) observed that underwater tail slaps occurred during all events with feeding activity and that no underwater tail slaps were detected during other behaviours such as socialising or travelling. If killer whales switched to a feeding behaviour that does not rely on underwater tail slap production, whether during day or night, such feeding events would remain undetected in our study. However, given known feeding behaviour of killer whales in Iceland when feeding upon herring, we are confident that recorded underwater tail slaps were good proxies of feeding activity within presence events. To compare the difference in the number of presence events with feeding activity (i.e. presence events with at least one underwater tail slap) between day and night, we used a generalised linear model (Zuur 2009) in R (package *stats*). Presence events with feeding activity (i.e. feeding events: 0 if no tail slap and 1 if at least one tail slap recorded) were used as the binomial response variable, and the explanatory variable was the day–night period:

$${\text{Glm}} \,\left({{\text{Feeding}}\;{\text{event}}\sim{\text{Light period}},{\text{ family}} = {\text{binomial}}\,\left({{\text{link}} = {\text{logit}}} \right)} \right)$$

We also used the same model to compare the difference in the number of presence events with at least one of each sound category between day and night:

$${\text{Glm}} \,\left({{\text{Sound}}\;{\text{category event}}\sim{\text{Light period}},{\text{ family}} = {\text{binomial}}\,\left( {{\text{link}} = {\text{logit}}} \right)} \right)$$

### Variations in sound production with feeding behaviour and day/night

To test whether sound production was related to underwater tail slaps (as a proxy of feeding activity), and whether there were differences between day and night, we used generalised linear models where the number of sounds of each category was the response variable and both the rate of underwater tail slaps per presence event, and the light period (as a categorical variable: day/night) were explanatory variables. Killer whale group composition during all presence events was unknown, thus we could not control for group identity in our analyses. Presence events were assumed to be statistically independent feeding bouts, either performed by the same or by a different group. As our response variable was a number per presence event, we used a Poisson distribution (with a *log* link function), and set the duration of each presence event as an offset, thus approximating a production rate (Zuur 2009). We then repeated the same model structure but added an interaction term between the two explanatory variables. We chose the better of the two models (with or without interaction) for each sound category based upon the Aikake Information Criterion (AIC) selection. Two models were considered different if their ΔAIC was higher than 2, in which case the lowest AIC defined the best model. However, if the ΔAIC was lower than 2, we selected the model with the lower degrees of freedom. In addition, we supported the models’ selection by conducting an ANOVA between the two models with and without the interaction term. We used the function *anova* in R, by setting a *χ* ^2^ test, which allowed us to test for a significant difference between the two models. To avoid type 1 error inflation with multiple tests in our interpretation of the 6 models (one per sound category, excluding tail slaps) we applied a Bonferroni correction, by dividing the significant *p* value threshold by 6, so that a factor had a significant effect if its *p* value was lower than 0.008.

## Results

### Acoustic processing

The EAR recorded a total of 5093 files, 47% during day and 46% during night. At the beginning of the recording period (22 February) sunrise occurred around 8:46 GMT (0) and sunset around 18:45 GMT (0), while at the end of the recording period (31 March) sunrise occurred around 6:30 GMT (0) and sunset around 20:22 GMT (0). From these files we extracted 3239 sounds from the first minute of 544 files (S1 Table), representing 11.5% of all recordings. From all the files with recorded sounds during the first minute, 59% were during the day, 34% during the night and 7% during twilight. Excluding recordings during twilight, we obtained 544 files with sounds, i.e. 10.7% of all recordings (63% during the day and 37% during the night).

We obtained similar mean RMS SPL of the ambient noise between day and night, with a mean difference of 1.71 ± 5.2 dB re 1 muPa^2^ between the two periods. Similarly, we found that 91% of herding calls (linear and nonlinear) recorded during the day and 87% of herding calls recorded during the night were of high quality, i.e. the signal-to-noise ratio in at least one 3rd-octave band was at least 10 dB. These results allowed us to consider that recording quality between day and night periods were similar. Given the high quality rate of recorded calls, we used the entire dataset without removing the lowest quality sounds, assuming that in the rare cases when lower-quality herding calls were detected, other sounds could also be representatively detected.

### Diel variation in sound production

**Fig. 3:**
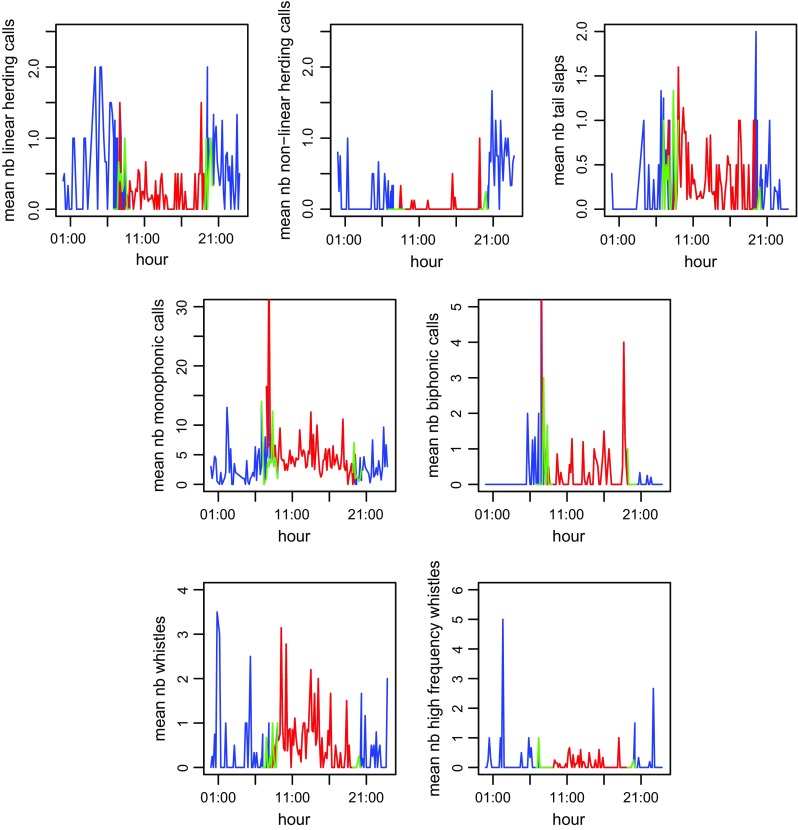
Mean number (nb) of all sound classes produced from all the recordings (37 days) plotted against time of day (hour). *Blue* indicates night-time, *red* indicates daytime and *green* indicates the twilight

From all the extracted sounds, we plotted mean numbers of sounds detected in each 1 min sample recorded every 10 min among a 24-h timeline (Fig. 3). This shows time of occurrence of killer whales acoustic encounters within the fjord. Interestingly, most of the sounds were produced at all times of day (Fig. 3), except for biphonic calls which were only produced during daytime and at the beginning (until 23:00 approximately) and the end of night-time (after 5:00 approximately). We also noticed a very low occurrence of nonlinear herding call during daytime and tail slap sounds during the middle of the night-time (Fig. 3). Observing raw data among a 24-h timeline revealed some diel trend, which we then tried to assess through presence events. Thus, we obtained 22 presence events during the day, with a mean duration of 18.7 ± 2.8 min per presence event, and 24 during the night, with a mean duration of 9.6 ± 2.8 min per presence event. Because we obtained a similar number of presence events between day (22) and night (24), but observed twice-longer durations during the day than night (*Difference* = 9.1 ± 3.9, *t* = 2.3, *p* = 0.03), we decided to use the rate of sound production for each sound category per presence event for all subsequent comparisons of sound production between day and night. These observations allowed us to compare characteristics of presence events between these two periods.

For both day and night all sound categories defined in this study were observed, but with different occurrence percentages (Table 1). Underwater tail slaps were recorded during 77% of the day events and during 50% of the night events, revealing feeding activity both during day and night (Table 1). Though the percentage of feeding events (i.e. presence events with at least one underwater tail slap) was greater during the day (77%,) than night (50%, Table 1), this difference was not statistically significant based on the generalised linear model (*z* = −1.88, *p* = 0.06). In addition, we observed that both during day and night, biphonic calls were produced less frequently than monophonic calls. Monophonic calls were produced in all but 2 presence events during night, whereas biphonic calls were used more often during day (50% of events, Table 1) than night (25% of events, Table 1) but this difference was non-significant (*z* = 1.38, *p* = 0.17). Similarly, whistles and high-frequency whistles were produced more often during day (86% and 59% of events, Table 1) than night (54% and 38% of events, Table 1), but the difference was only significant for whistles (*z* = 2.19, *p* = 0.03) and not for high-frequency whistles (*z* = 1.02, *p* = 0.3). Conversely, production of both linear and nonlinear herding calls occurred more frequently at night (71% and 58% of events, see Table 1) than during day (50 and 23% of events, Table 1), but the difference was non-significant for linear herding calls (z = −1.87, p = 0.06) and significant for nonlinear herding calls (z = −2.65, *p* = 0.008).

**Table 1:** Percentages (and number) of presence events with at least one instance of each sound type

Sounds category	Linear herding call	Nonlinear herding call	Tail slaps	Monophonic	Biphonic	Whistles	High-frequency whistles
Day (22) (number of presence events)	50% (11)	23% (5)	77% (17)	100% (22)	50% (11)	86% (19)	59% (13)
Night (24) (number of presence events)	71% (17)	58% (14)	50% (12)	92% (22)	25% (6)	54% (13)	38% (9)

**Fig. 4:**
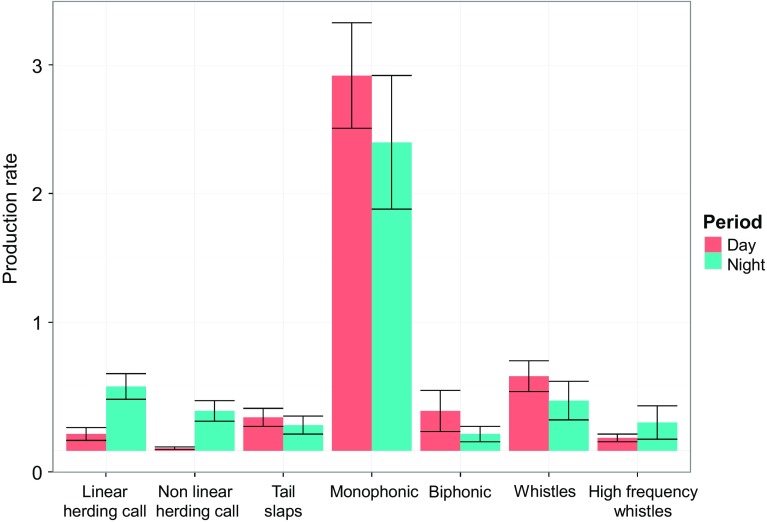
Mean rate of sound production (number of sounds per min) of different sound categories during day and night. Note that ‘Tail slaps’ are acoustic cues of feeding activity, whereas all other sounds are produced by the killer whales directly as acoustic signals

Even when corrected for presence event duration, sound production rates followed the same pattern as described above, except for high-frequency whistles (Fig. 4). High-frequency whistles were produced at slightly higher rates at night than during the day, despite being produced in more events during day than night (Table 2).

**Table 2:** Results of the generalised linear models, explaining the different sound categories in relation to tail slap rate and the light period per event, with or without interaction (*Rate of tail slap:Night)* and using event duration as an offset

Response variable	Explanatory variables	Estimate	*Z*-value	*P*
Linear herding calls	Rate tail slap	**1.37**	**6.55**	<**0.001**
	Night	**1.50**	**9.86**	<**0.001**
Nonlinear herding calls	Rate tail slap	−0.09	−0.19	0.85
	Night	**2.95**	**8.83**	<**0.001**
Monophonic calls	Rate tail slap	**0.78**	**9.49**	<**0.001**
	Night	−0.10	−1.54	0.12
	Interaction (*Rate tail slap:Night*)	−**0.62**	−**3.17**	**0.002**
Biphonic calls	Rate tail slap	0.26	0.76	0.45
	Night	−**1.15**	−**3.96**	<**0.001**
	Interaction (*Rate tail slap:Night*)	**2.01**	**3.74**	<**0.001**
Whistles	Rate tail slap	**1.13**	**0.20**	<**0.001**
	Night	−0.19	−1.03	0.30
	Interaction (*Rate tail slap:Night*)	−1.09	−1.88	0.06
High-frequency whistles	Rate tail slap	0.97	2.01	0.04
	Night	**1.34**	**4.43**	<**0.001**
	Interaction (*Rate tail slap:Night*)	−**5.73**	−**3.52**	<**0.001**

The base level for the categorical variable ‘*Period’* is ‘*Day’*. Thus, the effect of the variable ‘*Rate of tail slap*’ was estimated for data where *Period* = *Day*, and the interaction (*Rate of tail slap:Night*) estimated the difference between the effects of the variables ‘*Rate of tail slap*’ for both categories, i.e. the effect during night minus the effect during day. We considered a fixed factor significantly related to the explained factor if the *P* value was below 0.008 (in bold), after applying a Bonferroni correction

### Correlation with feeding behaviour

Based upon AIC criteria and the ANOVA tests, we found that the models without interaction between the rate of underwater tail slaps and the light period better explained the production of herding calls (both linear and nonlinear), whereas for all the other sound categories the models using the interaction term were selected (S2 Table). We observed that the production of linear herding calls was significantly and positively related to the rate of underwater tail slaps, consistent with the hypothesis of the herding role of this call just before slapping the herring schools increasing feeding efficiency. However, for nonlinear herding calls no correlation was found with the rate of underwater tail slap production. As for the light period, we observed that during the night the numbers of herding calls (both linear and nonlinear) were significantly higher than during the day, for a given rate of underwater tail slap (Table 2).

The number of biphonic calls was significantly lower during the night than during the day; however, their production was significantly associated to the rate of underwater tail slap at night but not during the day (Table 2). In contrast, we observed that during the day monophonic calls and whistles were significantly and positively related to the rate of underwater tail slaps, while during the night whistles showed no correlation whereas monophonic calls were still positively related to the rate of underwater tail slap but with a much lower relationship than during the day (Table 2). Finally, high-frequency whistles were produced more often during night and had a significantly negative relationship to the rate of underwater tail slap, which was not observed during the day (Table 2).

## Discussion

Remote acoustic monitoring of killer whale sounds showed for the first time that Icelandic killer whales fed roughly equally both during the day and night, using underwater tail slaps as acoustic markers of feeding activity. Comparisons of sound production during day and night showed significant diel variation in acoustic behaviour, previously undocumented in herring-eating killer whales. Acoustics is the main communication channel in killer whales, so the pronounced diel variation in production of different sound categories suggests underlying changes in behaviour.

Using underwater tail slaps as a direct indicator of feeding, we observed that killer whales foraged during 77% of the day presence events and 50% of the night events. The overall difference of these percentages of feeding events between day and night was not significant (*p* = 0.06), albeit close to significance at 0.05. This suggests that killer whales foraged at night to a similar extent as during the day; however, we cannot rule out that significant differences could be identified with an increased sample size.

Marine mammals are adapted for low-light conditions (Peichl et al. 2001) and use acoustic senses to their maximum advantage, such as in localising prey (Norris 1968). Night-time foraging is common and often advantageous because many prey species come closer to the surface at night and are less likely to detect predators (Norris et al. 1994; Thomas and Thorne 2001; Plötz et al. 2001; Benoit-Bird and Au 2003).

For instance, several studies using bio-loggers revealed diel foraging variation in southern elephant seals, as they dove at shallower depth during the night than during daylights hours (Hindell et al. 1991; Biuw et al. 2007; Guinet et al. 2014), suggesting a migration of seals’ prey, the myctophids, to a shallower depth. Indeed, light level is most likely to induce the vertical distribution of myctophids, since southern elephant seals avoid layers in the water column where the light intensity is too high during daytime foraging as well (Jaud et al. 2012). Similarly, deploying tags on long finned pilot whales, Baird et al. (2002) revealed diel variation of pilot whales foraging behaviour feeding on squid, but in contrast they observed very shallow dives during the day but deep dives at night. This was presumably because the whales could only hunt at night when squid came closer to the surface and spent daytime hours resting or socialising at the surface. Similar studies have also been conducted among baleen whales. For example, Friedlaender et al. (2009) observed that North West Atlantic humpback whales fed at the surface during the day, whereas at nigh they fed near the bottom, which correlated with the diel migration of their prey, the sand lance.

Predators that target herring in high latitudes during winter, when daylight is very short, such as killer whales, likely face selective pressures to adjust their foraging strategies to successfully capture their prey despite changes in the prey’s behaviour. Variations in herring schooling behaviour depending on light availability (Blaxter and Batty 1987) may lead to changes in their predators’ foraging strategies. We found that killer whales produced sounds from every category during feeding events during both day and night, but that the production of linear herding calls was higher at night and positively associated with the rate of underwater tail slaps. This result is in agreement with previous suggestions of the function of herding calls to herd the herring (Simon et al. 2006). The lack of light at night may make it more difficult to herd herring into schools because killer whales cannot use their white undersides to scare and herd the fish as they do during the day (Similä and Ugarte 1993). Thus, killer whales may significantly increase the production of herding calls at night to deal with the lack of light as tools to assist the herding of herring. However, we also noticed a short period during the night (between midnight and 3 am) that killer whale have produced some linear herding call without any tail slap production. This absence of co-occurrence between both sounds may reveal a feeding failure. During the middle of the night, at the darkest period, herring might be more disperse (Blaxter and Batty 1987), making killer whales’ foraging harder.

Variation in daytime vs night-time acoustic behaviour during feeding can be related not only to the amount of light, but also to differences in herring behaviour. Herring perform diel vertical migrations, rising closer to the surface at night (Dommasnes et al. 1994; Huse and Ona 1996). Our study area was rather shallow (max depth about 40 m), but still the diel variation in the depth distribution of prey may have caused changes in the hunting tactics and therefore acoustic behaviour of killer whales. Variations in herring schooling behaviour depending on light conditions have also been reported: herring was less active and less likely to form schools in darkness (Blaxter and Batty 1987), which may affect killer whale foraging tactics and calling behaviour. If so, the effort to herd herring might increase during night-time with an increase in herding call production in order to stimulate the anti-predatory schooling behaviour of the fish.

In contrast to the typical ‘herding’ calls, nonlinear herding calls were produced without any relation to the underwater tail slap rate, suggesting that they might have a different function. Nonlinear herding calls might be more effective than linear herding calls in herding herring in the absence of light, as they reach a larger range of frequencies that could match herring of diverse body sizes and swim-bladder resonant frequencies. In other species, nonlinear calls are produced predominantly by specific age-classes, such as juveniles, and can be a non-adaptive by-product of the physics of the sound production mechanism (Fitch et al. 2002). Future work will be necessary to investigate if this is the case in killer whales as well.

As killer whales were acoustically active and foraged both at night and during the day, we assessed how each sound category was associated with feeding context and whether it was used similarly during the day and at night. At night biphonic calls were positively related to feeding attempts, while monophonic calls were positively correlated with the rate of underwater tail slaps during the day. These results are in agreement with previous studies, which showed that killer whales have high rates of sound production during feeding (Simon et al. 2007; Samarra and Miller 2015). Day-night variation in correlation with underwater tail slap rate may suggest different functional roles of these sound categories. In the North Pacific fish-eating killer whales, biphonic calls have higher source levels (Miller 2006) and are more directional (Miller 2002) than monophonic calls. Together with their increased usage in the contexts of pod mixing (Filatova et al. 2009, 2013), this suggests that biphonic calls are used to track the position of family members, while monophonic calls are close-range intra-group contact signals (Filatova et al. 2009). Icelandic killer whales produced more biphonic calls during the day than during night, but they were related to the underwater tail slap rates only during the night. This result could reflect the possibility that their directionality allowed the whales to acoustically track the orientations and movements of each other in darkness in the context of a coordinated hunt (Miller 2002; Lammers and Au 2003). Although herring-eating killer whales in Iceland also increase the use of biphonic calls during daytime feeding (Samarra and Miller 2015), their use in other behavioural contexts in our dataset may have explained the lack of a significant relationship with tail slap rate.

Whistles appeared positively correlated with the rate of underwater tail slaps both during the day and night. Whistles are characterised by high frequencies and low sound pressure levels (Miller 2006), and so are considered to be important in close-range communication, such as during social interaction (Riesch et al. 2006, 2008). Simon et al. (2007) also showed increased whistle production during feeding activity for Icelandic killer whales. Therefore, we could assume that whistles may play a role in coordinated foraging.

In Iceland, killer whales are acoustically active while foraging and socialising but not while travelling (Simon et al. 2007; Samarra and Miller 2015). Thus, during “non-feeding” activities (i.e. presence events with calls and/or whistles but no underwater tail slaps) killer whales were most likely to be socialising, but without any acoustic marker we cannot confirm any behaviour. Only high-frequency whistles produced at night appeared to be possibly specific to the “non-feeding” activity. However, this has to be interpreted with caution since it is likely that our sample of high-frequency whistles is not representative of the entire repertoire produced due to sampling frequency constraints.

Acoustic markers of feeding behaviour (such as echolocation or buzz production) allow for the monitoring of diel foraging behaviours. Many studies have revealed increases in foraging activity at night for odontocetes, based upon passive acoustic monitoring (e.g. harbour porpoises: Todd et al. 2009; Yangtze finless porpoises: Wang et al. 2014; beaked whales: McDonald et al. 2009; deep diving odontocetes in Hawaii: Au et al. 2013). Indeed, for species that produce feeding-specific sounds, passive acoustic monitoring can be an extremely useful tool to understand habitat use, diel and seasonal behavioural patterns. Here, we show that acoustic markers of feeding activity produced by herring-eating killer whales can be reliably used for passive acoustic monitoring.

In conclusion, we have revealed that night-time foraging occurs in herring-eating killer whales and likely represents a substantial amount of killer whale food intake during winter in Iceland. This contrasts with reports for other fish-eating killer whales that appear to forage mostly during the day, with reduced activity levels at night (Baird et al. 2005). Our study brings new evidence of the importance of night-time foraging, suggesting that detailed research into this behaviour is essential to fully understand predator–prey relationships, and that passive acoustic monitoring is a powerful tool to more fully assess these interactions. Our results indicate that Icelandic killer whales have adapted their diel feeding activity to optimise their foraging success.

## Electronic supplementary material

Below is the link to the electronic supplementary material.

Supplementary material 1 (PDF 239 kb)
