# Supplementary material for: Icelandic herring-eating killer whales feed at night
Source: Mar Biol. 2017 Jan 30;164(2):32. doi: 10.1007/s00227-016-3059-8 (PMC5281646; doi:10.1007/s00227-016-3059-8)
Supplement: Supplementary file 1 — Supplementary material 1 (PDF 239 kb) [file 227_2016_3059_MOESM1_ESM.pdf]

Icelandic herring-eating killer whales feed at night

Gaëtan Richard[1,2,a], Olga A. Filatova[1,3], Filipa I. P. Samarra[1,4], Ivan D. Fedutin[3], Marc Lammers[5], and Patrick J. Miller[1]

[1] Sea Mammal Research Unit, School of Biology, University of St Andrews, St Andrews, Fife KY16 8LB, UK

[2] Ecole Normale Supérieure de Lyon, Master BioSciences, Lyon, 69007 France

[3] Faculty of Biology, Moscow State University, Moscow 119234, Russia

[4] Marine and Freshwater Research Institute, Skulagata 4, 101 Reykjavík, Iceland

[5] Hawaii Institute of Marine Biology, Kaneohe, HI 96744, USA

[a] gaetan-gs.richard@laposte.net

Supplementary material

Sounds category	Linear herding calls	Non-linear herding calls	Tail slaps	Monophonic calls	Biphonic calls	Whistles	High frequency whistles	Total sounds	Number of files with sounds
**Day rate (number)**	0.13±0.05 (66)	0.02±0.01 (10)	0.26±0.07 (125)	2.92±0.41 (1653)	0.31±0.16 (110)	0.58±0.12 (236)	0.10±0.03 (43)	4.33±0.56 (2244)	345
**Night rate (number)**	0.50±0.10 (127)	0.31±0.08 (90)	0.20±0.07 (44)	2.40±0.52 (597)	0.13±0.06 (33)	0.39±0.15 (68)	0.22±0.13 (33)	4.14±0.53 (995)	199
Global rate (number)	*0.31±0.06 (193)*	*0.16±0.04 (100)*	*0.23±0.05 (169)*	*2.67±0.33 (2250)*	*0.22±0.09 (143)*	*0.49±0.10 (304)*	*0.16±0.06 (76)*	*4.24±0.38 (3239)*	*544*

S1 Table. Comparison of the mean rate of sound production (number of sounds per min) with the standard errors, and number of sounds for each category between day, night and both. The number of files is also the total amount of recording time (min) used in the study.

Response variable	Models compared	AIC (df)	ΔAIC	ANOVA: p-value χ^2 test
**Linear herding calls**	No interaction<br>Interaction	269.8 (df=3)<br>271.4 (df=4)	1.6	p=0.5
**Non-linear herding calls**	No interaction<br>Interaction	188.9 (df=3)<br>190.7 (df=4)	1.8	p=0.6
**Biphonic calls**	No interaction<br>Interaction	333.6 (df=3)<br>323.2 (df=4)	**10.4**	p<0.001
**Monophonic calls**	No interaction<br>Interaction	1287.5 (df=3)<br>1278.7 (df=4)	**8.8**	**p=0.001**
**Ultrasonic whistles**	No interaction<br>Interaction	183.3 (df=3)<br>167.4 (df=4)	**15.9**	**P<0.001**
**Whistles**	No interaction<br>Interaction	431.9 (df=3)<br>429.7 (df=4)	**2.2**	**p=0.04**

S2 Table: Selection between models with and without an interaction term between the explanatory variables (rate of tail slap and light period) for each sound category. Two methods are represented: 1) one based on the Akaike Information Criterion, with bold representing a ΔAIC>2, and; 2) one based on an ANOVA using χ^2 test, with statistically significant differences ($p<0.05$) in bold.
